# Carbachol-Mediated Endocytosis of NHE3 Involves a Clathrin-Independent Mechanism Requiring Lipid Rafts and Cdc42

**DOI:** 10.1159/000358659

**Published:** 2014-03-28

**Authors:** Nicholas C. Zachos, Bharath Alamelumangpuram, Luke J. Lee, Peng Wang, Olga Kovbasnjuk

**Affiliations:** Department of Medicine/Gastroenterology and Hepatology, Johns Hopkins University School of Medicine, Baltimore, MD, USA

**Keywords:** Protein trafficking, Intestinal epithelial cells, Intracellular calcium

## Abstract

**Background:**

In intestinal epithelial cells, acute regulation of the brush border Na^+^/H^+^ exchanger, NHE3, usually occurs by changes in endocytosis and/or exocytosis. Constitutive NHE3 endocytosis involves clathrin. Carbachol (CCH), which elevates intracellular Ca^2+^ ([Ca^2+^]_i_), decreases NHE3 activity and stimulates endocytosis; however, the mechanism involved in calcium-mediated endocytosis of NHE3 is unclear. A pool of NHE3 resides in lipid rafts, which contributes to basal, but not cAMP-mediated, NHE3 trafficking, suggesting that an alternative mechanism exists for NHE3 endocytosis. Cdc42 was demonstrated to play an integral role in some cases of cholesterol-sensitive, clathrin-independent endocytosis. Therefore, the current study was designed to test the hypotheses that (1) clathrin-mediated endocytosis (CME) is involved in constitutive, but not CCH-mediated, endocytosis of NHE3, and (2) CCH-mediated endocytosis of NHE3 occurs through a lipid raft, activated Cdc42-dependent pathway that does not involve clathrin.

**Methods:**

The role of Cdc42 and lipid rafts on NHE3 activity and endocytosis were investigated in polarized Caco-2/BBe cells using pharmacological and shRNA knockdown approaches.

**Results:**

Basal NHE3 activity was increased in the presence of CME blockers (chlorpromazine; K^+^ depletion) supporting previous reports that constitutive NHE3 endocytosis is clathrin dependent. In contrast, CCH-inhibition of NHE3 activity was abolished in Caco-2/BBe cells treated with MβCD (to disrupt lipid rafts) as well as in Cdc42 knockdown cells but was unaffected by CME blockers.

**Conclusion:**

CCH-mediated inhibition of NHE3 activity is not dependent on clathrin and involves lipid rafts and requires Cdc42.

## Introduction

The BB Na^+^/H^+^ Exchanger, NHE3, accounts for most of the electroneutral Na^+^ absorption that occurs during digestion. NHE3 is initially inhibited in digestion and later in digestion is stimulated [1]. In most diarrheal diseases, there is inhibition of neutral NaCl absorption in the Na^+^ absorptive cell primarily due to NHE3 inhibition. In the intact ileum, carbachol (CCH) elevated intracellular calcium ([Ca^2+^]_i_) via basolateral membrane (BLM) muscarinic receptors (M3) which inhibited NHE3 activity by 40% and similarly decreased surface expression of NHE3 [2–5]. NHE3 is often regulated by changes in its plasma membrane versus intracellular location as a result of changes in the rates of endocytosis and/or exocytosis [6–14]. Basal NHE3 endocytosis occurs by a clathrin-dependent process which partially involves lipid rafts [15, 16]. Previous studies demonstrated that constitutive and cAMP regulated NHE3 endocytosis occurs through a clathrin-dependent process [9, 17]. Carbachol elevation of [Ca^2+^]_i_ decreases NHE3 activity and stimulates endocytosis as early as one minute; however, studies examining [Ca^2+^]_i_-mediated endocytosis of NHE3 in polarized intestinal epithelial cells are incomplete [14]. A pool of BB NHE3 resides in lipid rafts which contributes to basal, but not cAMP-mediated, NHE3 trafficking, suggesting an alternative mechanism exists for NHE3 endocytosis [15, 16]. Recently, the Rho GTPase, Cdc42, has been demonstrated to play an integral role in cholesterol-sensitive, clathrin-independent endocytosis of integral membrane proteins including the folate receptor as well as the *Helicobacter pylori* VacA cytotoxin [18–20]. Cdc42 is necessary for a clathrin-independent endocytic process termed the CLathrin Independent Carrier (CLIC)/GPI-anchored protein-enriched Early Endocytic Compartment (GEEC) pathway [21, 22]. Previous studies designed to understand the role of elevated [Ca^2+^]_i_ on NHE3 trafficking have used non-physiologic agents including calcium ionophores and thapsigargin in fibroblast or non-polarized epithelial cells [17, 23–25]. This study tested the hypotheses that: (1) inhibition of clathrin-mediated endocytosis (CME), prevents basal but not CCH-mediated, endocytosis of NHE3, and (2) CCH-mediated endocytosis of NHE3 occurs through a lipid raft and activated Cdc42-dependent pathway that does not involve clathrin.

## Materials and Methods

### Reagents

Carbachol (CCH), chlorpromazine (CPZ), methyl-β-cyclodextrin (MβCD), and BAPTA-AM were from Sigma. Pirl-1 (8-cyclohexil-5,6-dihidro-4H-pyrazino[3,2,1-jk]carbazole), which inhibits guanine nucleotide exchange on Cdc42, was from Chembridge (San Diego, CA) [26]. AlexaFluor 488 conjugated wheat germ agglutinin (WGA) was from Invitrogen.

### Antibodies

Affinity-purified rabbit polyclonal antibodies to Cdc42 and clathrin heavy chain were from Cell Signaling. Polyclonal caveolin-1 antibody was from Santa Cruz. Unconjugated monoclonal anti-HA antibody was from Covance. AlexaFluor 594 conjugated anti-HA antibody was from Invitrogen.

### Cell Line

Caco-2/BBe cells express all four members of the NHERF gene family and small amounts of NHE3 [27]. Triple HA-tagged rabbit NHE3 was transiently expressed by adenovirus into Caco-2/BBe cells for transport and biochemical analysis. Caco-2/BBe cells were grown on Anapore filters (Nunc) until post-confluent for 12 days in Dulbecco’s modified Eagle’s medium supplemented with 25mM NaHCO_3_, 10mM HEPES, 0.1mM nonessential amino acids, 50 units/ml penicillin, 50 μg/ml streptomycin, and 10% fetal bovine serum in a 5% CO_2_, 95% air incubator at 37°C. Cells were serum starved overnight and then treated with 6mM EGTA for 2 h at 37°C. Caco-2/BBe cells were then exposed to 3HA-NHE3 adenovirus for 6 h at 37°C. Cells were allowed to recover in normal media over the next 40 h before study [28, 29].

### shRNA knockdown (KD) of Cdc42

5 shRNA constructs for Cdc42 from Open Biosystems were obtained through the Johns Hopkins University School of Medicine High Throughput Biology Center (HiT Center). shRNA constructs were packaged into lentivirus particles using HEK293A cells. Caco-2/BBe cells were infected with shRNA-containing lentiviruses and KD cells were selected using puromycin as a selection marker. Efficiency of KD was determined by Western blot. shRNA constructs that did not exhibit any measure of Cdc42 KD were used as negative controls.

### Measurement of Na^+^/H^+^ Exchange

Cellular Na^+^/H^+^ exchange activity in Caco-2/BBe cells grown to 14-days post-confluency on Transwell filters was determined fluorometrically using the intracellular pH-sensitive dye, 2,7-bis(carboxyethyl)5-6-carboxyfluoresceinacetoxy-methy ester (BCECF- AM, 5μM; Molecular Probes, Eugene, OR), as described previously [30]. Caco-2/BBe cells were exposed to 50mM NH4Cl during a 45-min dye loading, as described previously [28, 29, 31]. Cells were perfused initially with TMA^+^ solution alone or with 10μM carbachol for 1–10min (138mM tetramethylammonium chloride, 5mM KCl, 2mM CaCl_2_, 1mM MgSO_4_, 1mM NaH_2_PO_4_, 25 mM glucose, 20 mM HEPES, pH 7.4), before being switched to Na^+^ solution (138mM NaCl instead of TMA^+^) for the Na^+^-dependent pH*_i_* recovery. At the end of each experiment, the fluorescence ratio was calibrated to pH*_i_* using the high potassium/nigericin method. Na^+^/H^+^ exchange activity data were calculated as the ratio of Na^+^-dependent changes in pH*_i_* over initial time (ΔpH/min) of Na^+^-dependent pH recovery using at least three coverslips per condition in a single experiment. NHE3 activity was measured in cells treated with either vehicle or CCH (10μM; 5min) in the presence or absence of CPZ (10μM; 20min), methyl-β-cyclodextrin (MβCD; 10mM; 20min), or Pirl-1 (20μM; 20min). NHE3 activity was measured in the presence of 50μM HOE-694 (concentration sufficient to inhibit all other NHE isoforms). Initial rates were analyzed by using Origin (Microcal Software) to determine statistical significance among individual experiments. Means ± S.E. were determined from at least three separate experiments.

### Immunofluorescence

Polarized Caco-2/BBe cells grown on Anapore filters were imaged under 4% paraformaldahyde fixed conditions. NHE3 and clathrin colocalization was determined using standard confocal microscopy (Zeiss 510 META). NHE3 surface expression was observed using monoclonal anti-HA antibody conjugated to AlexaFluor 594 (1:100 dilution). The amount of surface NHE3 was compared against FITC-labeled wheat germ agglutinin (WGA) labeled Caco-2/BBe cell brush border (labeled at 4°C).

### Co-immunoprecipitation

Co-immunoprecipitation was performed as previously described [29]. Briefly, Caco-2/BBe cells were grown on 10-cm^2^ Transwell Petri dishes until 12 days post-confluence. On day 12, cells were transduced with the adenovirus 3HA-NHE3 construct as described above. On day 14, cells were serum-starved for 4 h and treated either with vehicle or 10 μm CCH for 5 min at 37 °C. Cells were washed three times in ice-cold PBS containing 50 mM Tris, scraped and lysed in 500 μl of ice-cold lysis buffer (10 mM HEPES, 50 mM NaCl, 5 mM EDTA, 1 mM benzamidine, 0.5% Triton X-100). Cell lysates were incubated with either anti-CHC or anti-caveolin- 1 antibodies conjugated to Protein G-agarose beads (Sigma) for 2 h with end-over-end rotation at 4 °C. Samples were washed five times with lysis buffer and immunoprecipitated proteins were eluted from beads with 2X sample buffer. Samples were resolved by 10% SDS-PAGE and proteins were detected with anti-HA (for NHE3), anti-CHC and anti-caveolin antibodies and visualized on an Odyssey Infrared Imaging System (Li-Cor, Lincoln, NE). Results were obtained from three individual experiments.

### NHE3 surface biotinylation

NHE3 surface biotinylation was performed as previously described [31]. Briefly, Caco-2/BBe cells (transiently expressing 3HA-tagged NHE3 adenovirus construct) monolayers were grown on Transwell filters (Corning). Cells treated with vehicle or CCH in the presence or absence of MβCD, CPZ or Pirl-1 were rinsed with ice-cold PBS and then incubated for 30min with gentle shaking in borate buffer (pH 8.0) with 1.25mg/ml NHS-SS-biotin (Pierce). After rinsing with PBS, cells were washed twice for 10 min with 100μM glycine to scavenge unreacted biotin and scraped into lysis buffer with protease inhibitors. Lysates were incubated with avidin-agarose beads (Pierce) to precipitate the biotinylated proteins. The beads were then washed and biotinylated proteins separated by SDS-PAGE and transferred to nitrocellulose membranes for quantitative Western blotting (Odyssey Infrared Imaging System) using anti-HA (for NHE3) antibody. Surface biotinylated NHE3 was compared to total NHE3 protein levels.

### Cdc42 actvation assay

Cell lysates were obtained from polarized Caco-2/BBe cells grown on Anapore filters for 14 days post-confluency. Caco-2/BBe cells were pretreated with vehicle or 35μM BAPTA-AM prior to exposure to 10μM CCH for various time points (1–60min). Epidermal growth factor (EGF) is a potent activator of Cdc42 and was used as a positive control [32]. Cdc42 activity (i.e. bound to GTP) was measured using Cdc42 Activation Assay Biochem Kit (Cytoskeleton) according to the manufacture’s protocol. Amount of activated Cdc42 was determined by Western blot analysis.

## Results

### Clathrin is not required for carbachol-mediated inhibition of NHE3 Activity in Caco-2/ BBe cells

**Fig. 1:**
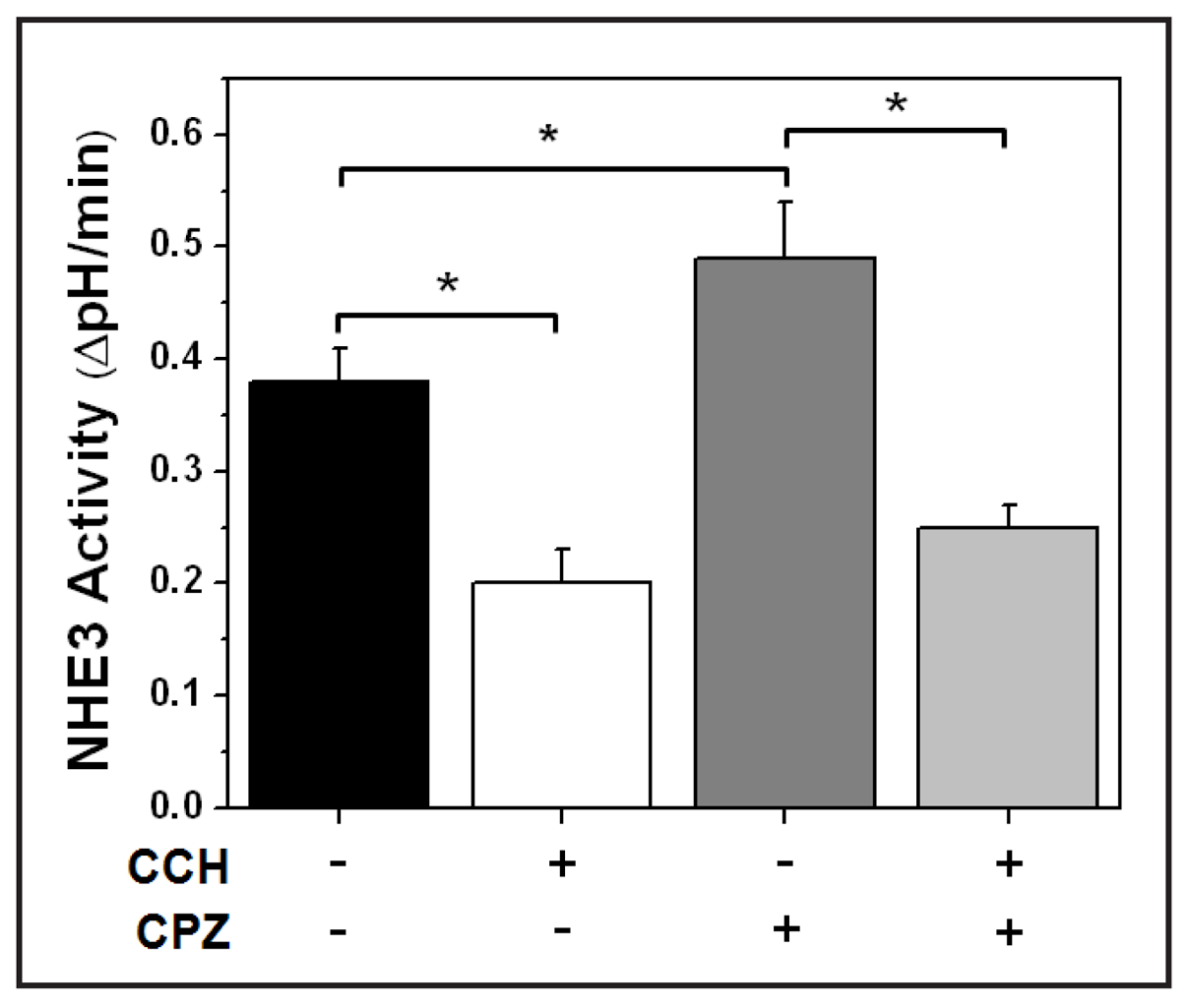
Clathrin is not required for carbachol-mediated inhibition of NHE3 Activity in Caco-/ BBe cells. NHE3 activity was measured by BCECF fluorimetry in differentiated Caco-2/BBe cells infected with adenovirus 3HA-NHE3 and exposed to either vehicle (*CTL = black bar*) or 10μM carbachol (*CCH*) in the presence or absence of the clathrin inhibitor, chlorpromazine (CPZ; 10μM). NHE3 activity was determined in the presence of 50 μM HOE-694 (concentration sufficient to inhibit all other NHE isoforms). CCH treatment resulted in a significant decrease of NHE3 activity in Caco-2/BBe/3HA-NHE3 cells in the presence and absence of CPZ for 30 minutes. In addition, CPZ treatment stimulated basal NHE3 activity in Caco-2/BBe cells by ~29%. *n* ≥ 4 for each condition. Rate of NHE3 activity (ΔpH/min) expressed as mean ± S.E. *p < 0.05 compared to untreated controls.

**Fig. 2:**
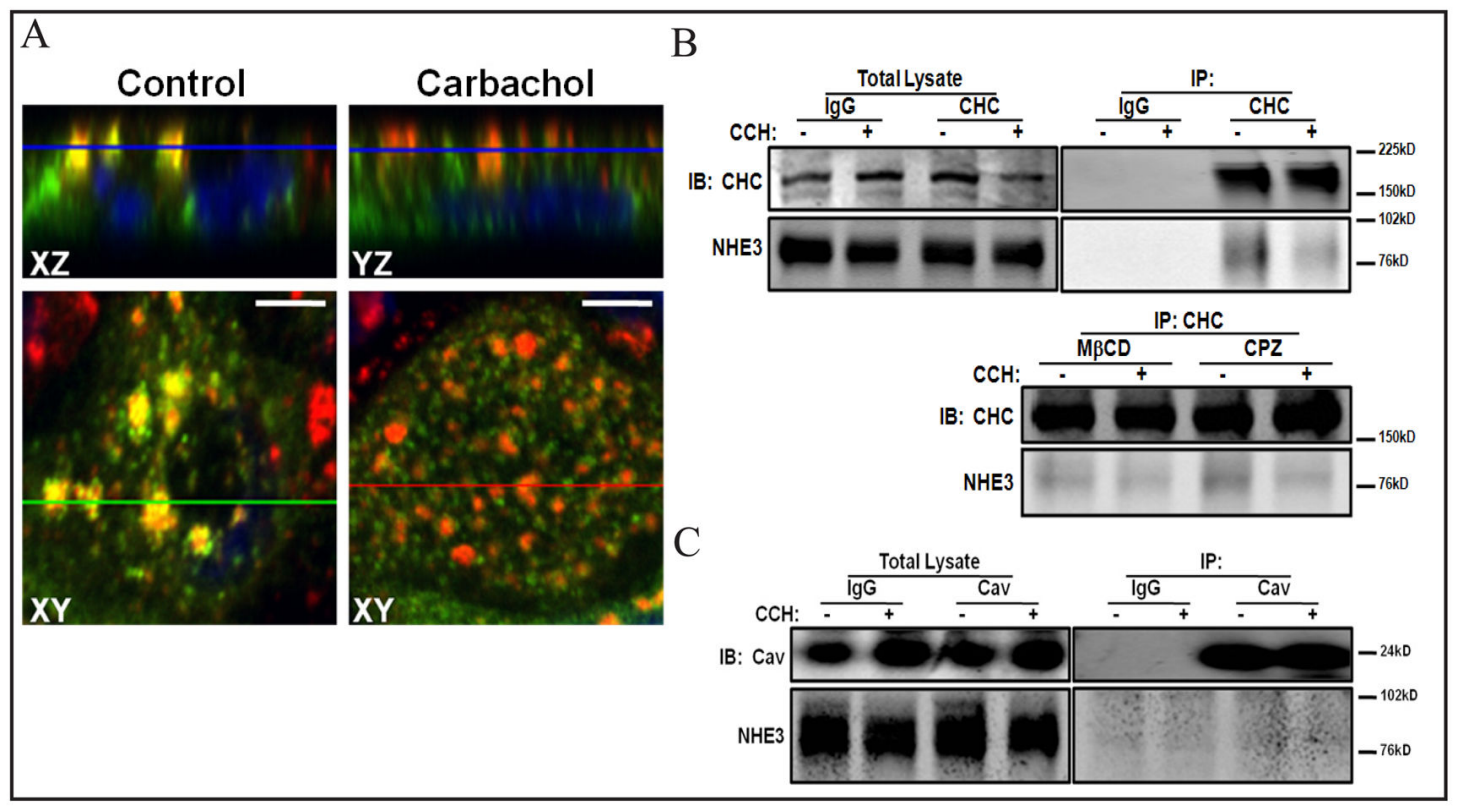
Basal association of NHE3 with clathrin is reduced after carbachol treatment. *A*, NHE3 colocalization with clathrin heavy chain was determined by confocal microscopy in Caco-2/BBe cells treated with CCH for 5 minutes. After treatment, cells were fixed and stained with antibodies to label NHE3 and clathrin. Under basal conditions, NHE3 (green) and clathrin (red) demonstrated partial colocalization in the apical domain of Caco-2/BBe cells. After CCH treatment, NHE3 and clathrin did not exhibit any colocalization in any region of Caco-2/BBe cells. Scale bar = 5μm. Images are representative of results from three independent experiments. *B-C*, Association of NHE3 with clathrin and caveolin-1 was determined by co-immunoprecipitation (co-IP) studies in Caco-2/BBe cells treated with CCH for 5 min. Immunoprecipitated clathrin associates with NHE3 under basal conditions; however, this interaction is decreased after 5 min CCH treatment. Similar results were observed after CCH treatment in cells pretreated with CPZ or MβCD. In contrast, NHE3 does not associate with caveolin-1 under any conditions in Caco-2/BBe cells. Rabbit polyclonal IgG antibody was used as a negative control. n = 3 independent experiments for each co-IP study.

Previous studies have demonstrated that constitutive NHE3 endocytosis occurs by a clathrin-dependent process [17, 23]. Caco-2/BBe cells were grown on filters until post-confluent for 12 days and were then infected with an adenovirus 3HA-NHE3 construct. NHE3 activity was determined 48 h after the infection. Caco-2BBe/3HA-NHE3 cells were pretreated with either vehicle or 10μM CPZ (blocks clathrin-coated pit formation) for 30min and then exposed to vehicle or CCH for 5min, prior to Na^+^-dependent recovery of pH*_i_* [33]. As shown in Figure 1, in vehicle pretreated controls, NHE3 activity was significantly inhibited (~47%) after CCH treatment (Fig. 1). In cells pretreated with CPZ, basal NHE3 activity was significantly increased by 29% compared to untreated controls (*p*<0.05) while CCH inhibition of NHE3 activity was unaffected by CPZ pretreatment (~49%). Similar results were obtained in cells exposed to K^+^-depleted conditions, which also blocks clathrin-mediated endocytosis by removing membrane-associated clathrin lattices (data not shown) [34]. These results suggest that basal NHE3 activity is, at least partially, regulated by clathrin-mediated endocytosis. However, CCH inhibition of NHE3 activity does not involve clathrin. Using immunofluorescence confocal microscopy, NHE3 and clathrin heavy chain did not appear to colocalize after CCH treatment but did exhibit some overlap under basal conditions (Fig. 2A). In order to demonstrate that CCH treatment alters the association of NHE3 and clathrin, we performed co-immunoprecipitation studies. As shown in Figure 2B, clathrin heavy chain (CHC) immunoprecipitated NHE3 under basal conditions and this association was reduced after 5 min CCH treatment. These results suggest that CCH-inhibition of NHE3 activity does not involve increased association with CHC. Furthermore, the association of NHE3 and CHC was not increased after CCH exposure in cells pretreated with CPZ or MβCD, which suggests that CCH inhibition of NHE3 activity involves a pool of NHE3 that does not associate with CHC. Moreover, we further demonstrated that caveolar-dependent endocytosis is also not involved in CCH regulation of NHE3 activity as immunoprecipiated caveolin-1 does not associate with NHE3 under basal or CCH treated conditions (Fig. 2C). Taken together, these results suggest that a non-clathrin, non-caveolar dependent pathway of endocytosis is involved in CCH-mediated inhibition of NHE3 activity. Previous studies have demonstrated that clathrin-independent endocytosis involves lipid rafts [21]. Furthermore, basal NHE3 activity is lipid raft dependent in OK cells [16] suggesting that an alternate pathway to clathrin-mediated endocytosis may exist for NHE3 trafficking. However, a role for lipid rafts in elevated [Ca^2+^] _i_ regulation of NHE3 activity has not been demonstrated. We therefore tested whether constitutive and/or CCH- inhibition of NHE3 activity involves lipid rafts.

### Lipid rafts are necessary for CCH-inhibition of NHE3 activity

**Fig. 3:**
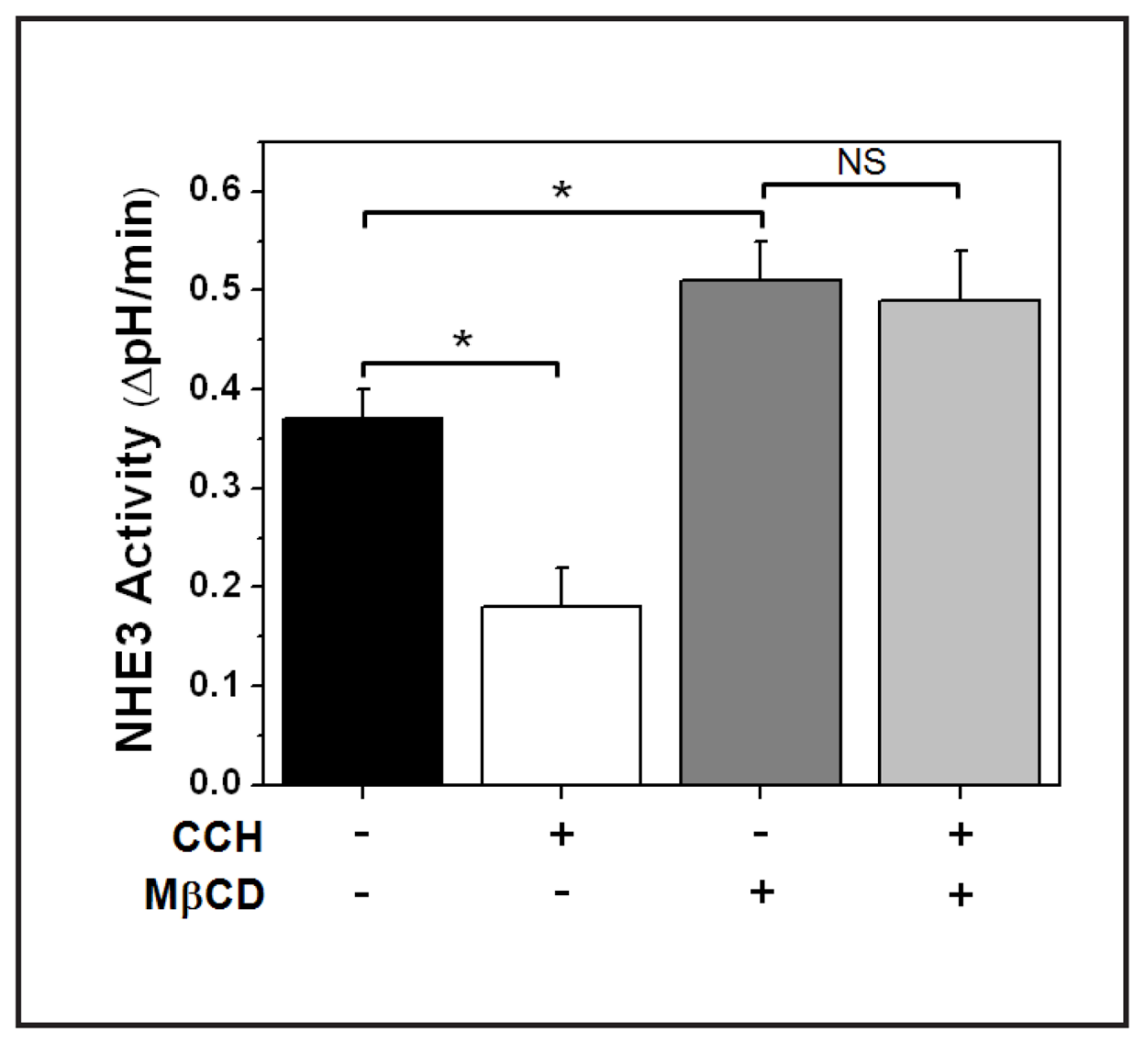
Lipid rafts are necessary for carbachol-inhibition of NHE3 activity. NHE3 activity was measured in Caco-2/BBe/3HA-NHE3 exposed to either vehicle (*CTL = black bar*) or 10μM carbachol (*CCH*) in the presence or absence of 10mM methyl-β-cyclodextrin (*MβCD*) pretreated for 20min. NHE3 activity was determined in the presence of 50 μM HOE-694 (concentration sufficient to inhibit all other NHE isoforms). CCH treatment resulted in a significant decrease of NHE3 activity in Caco- 2/BBe/3HA-NHE3 cells. In contrast, inhibition of NHE3 activity by CCH was completely abolished in Caco-2/BBe/3HA-NHE3 cells exposed to MβCD. MβCD also significantly stimulated basal NHE3 activity in Caco-2/BBe cells by ~30%. *n* ≥ 5 for each condition. Rate of NHE3 activity (ΔpH/min) expressed as mean ± S.E. *p<0.05 compared to untreated controls. NS = Not significant.

We determined the role of lipid rafts in basal and CCH inhibition of NHE3 activity using the cholesterol depleting agent, methyl-β-cylcodextrin (MβCD). In Caco-2/BBe cells pretreated with MβCD, basal NHE3 activity was stimulated by 30% (Fig. 3). This result suggests that a portion of BB NHE3 is associated with lipid rafts and that this fraction participates in constitutive endocytosis since disruption of lipid rafts results in increased NHE3 activity. This result is supported by surface biotinylation studies where BB NHE3 levels are increased by ~24% after CCH exposure in MβCD-treated cells (data not shown). In contrast to pretreatment with CPZ, disruption of lipid rafts by MβCD treatment completely abolished CCH inhibition of NHE3 activity (Fig. 3). Our current results in Caco-2/BBe cells support previous studies which demonstrated that a pool of BB NHE3 resides in lipid rafts in intestinal epithelial cells. Furthermore, we demonstrate that MβCD treatment completely prevented CCH inhibition of NHE3 activity suggesting that the pool of BB NHE3 inhibited by CCH is in lipid rafts (Fig. 3). Since all clathrin-independent endocytosis has been shown to involve lipid rafts and depend on small GTPases, we next tested whether CCH inhibition of NHE3 activity required the Rho GTPase, Cdc42 [35].

### Cdc42 is necessary for CCH-inhibition of NHE3 activity

**Fig. 4:**
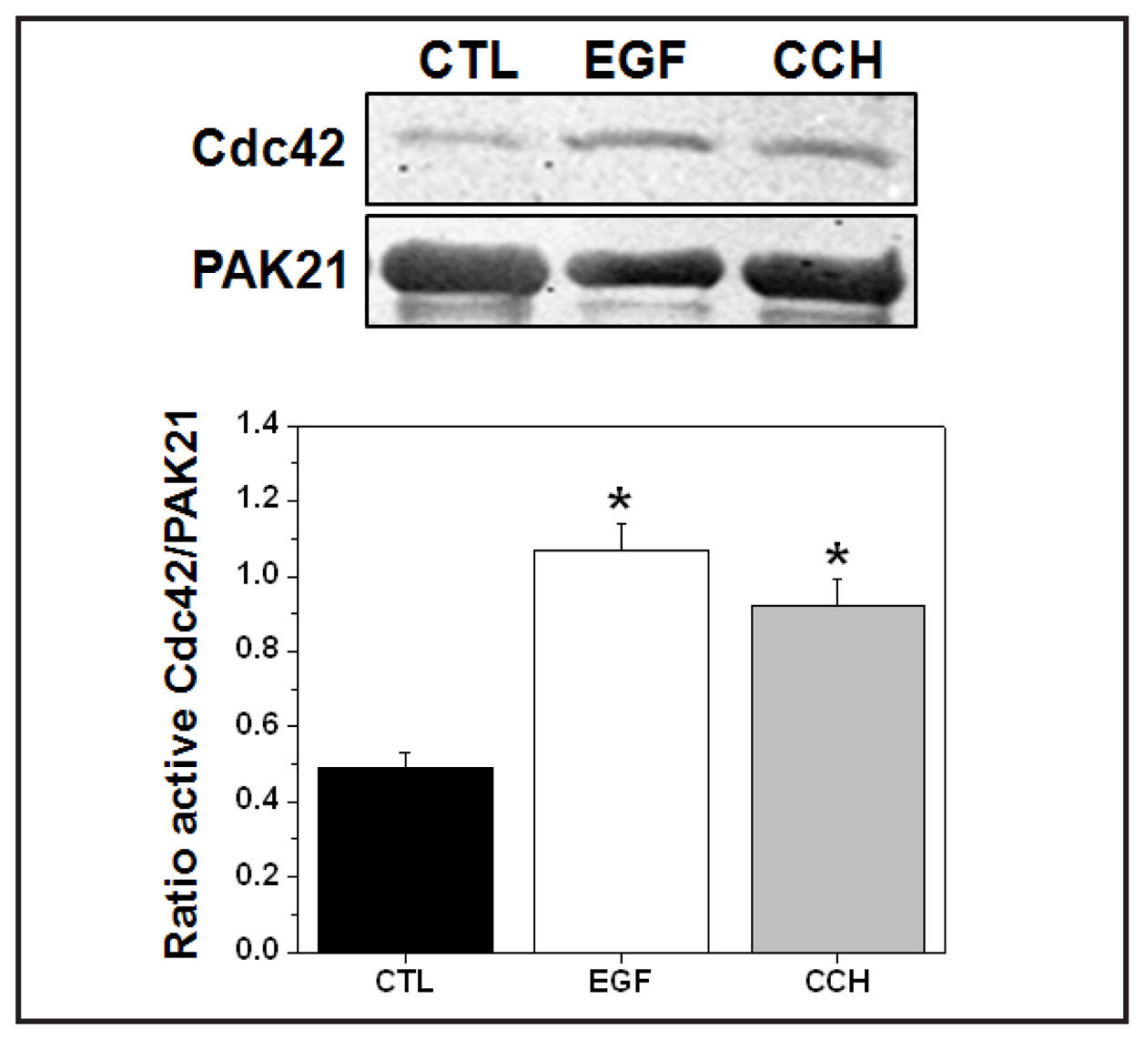
Carbachol signaling increases the amount of GTP-bound Cdc42 in Caco-2/BBe cells. The amount of GTP-bound Cdc42 under basal (*CTL*) and carbachol (*CCH*) treated conditions in Caco-2/ BBe cells was measured by pull-down assay using purified PAK21 binding protein. Caco-2/BBe cells were untreated or treated with CCH for 5 min, cells washed in ice cold PBS, and cell lysates collected. GTP-bound Cdc42 (i.e. activated Cdc42) was precipitated from cell lysates with PAK21 binding protein and visualized by western blot. 10μM CCH treatment significantly increased the amount of GTP-bound Cdc42 compared to controls. Treatment of Caco-2/BBe cells with EGF served as a positive control. Blots represent results obtained from three independent experiments. Cdc42 protein levels normalized to PAK21 expressed as mean ± S.E. *p<0.05 compared to controls.

**Fig. 5:**
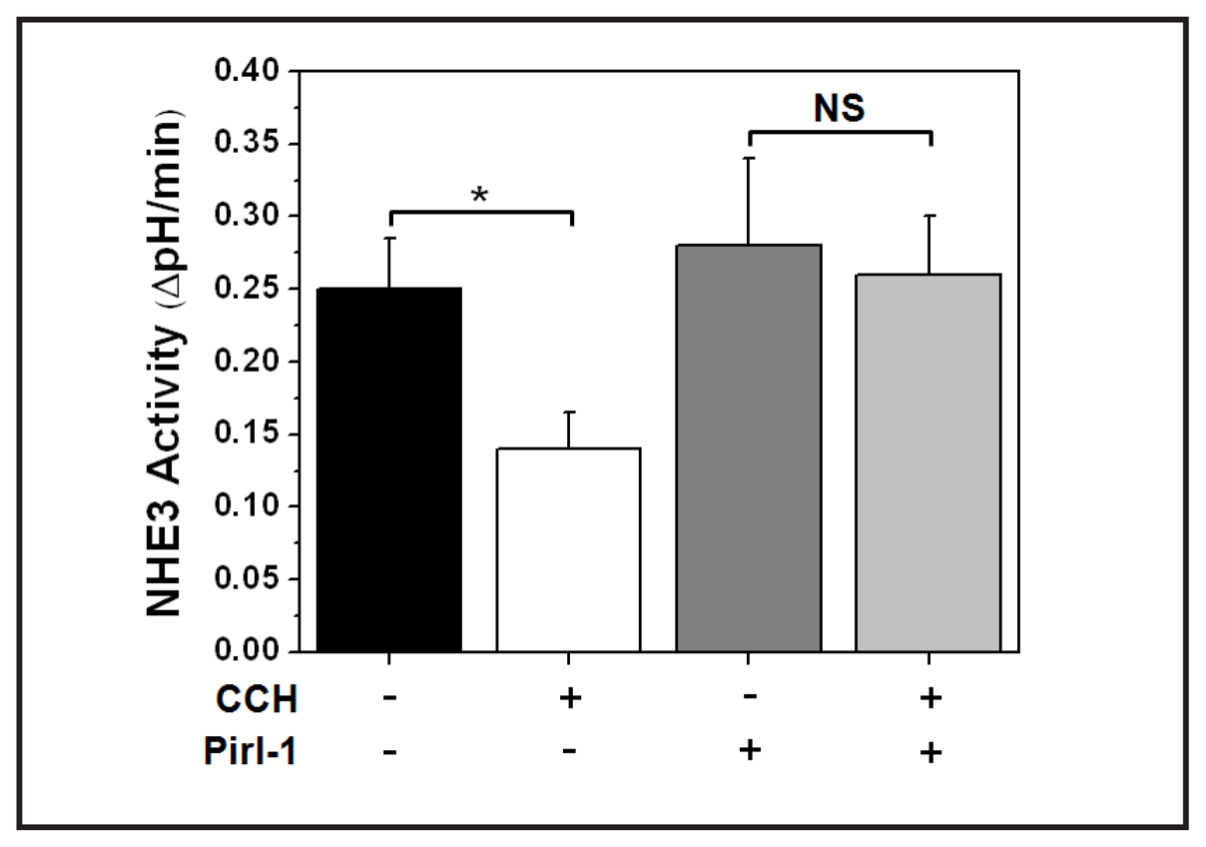
Cdc42 is necessary for carbachol-inhibition of NHE3 activity. NHE3 activity was measured in Caco-2/BBe/3HA-NHE3 exposed to either vehicle (*CTL = black bar*) or 10μM carbachol (*CCH*) in the presence or absence of the Cdc42 specific inhibitor, Pirl-1 (20μM; pretreated for 30min. NHE3 activity was determined in the presence of 50 μM HOE-694 (concentration sufficient to inhibit all other NHE isoforms). CCH treatment resulted in a significant decrease of NHE3 activity in Caco-2/BBe/3HA-NHE3 cells. In contrast, inhibition of NHE3 activity by CCH was completely abolished in Caco-2/BBe/3HA-NHE3 cells exposed to Pirl-1. Exposure of cells to Pirl-1 did not alter basal NHE3 activity in Caco-2/BBe cells. *n* ≥ 3 for each condition. Rate of NHE3 activity (ΔpH/min) expressed as mean ± S.E. *p<0.05 compared to untreated controls. NS = Not significant.

**Fig. 6:**
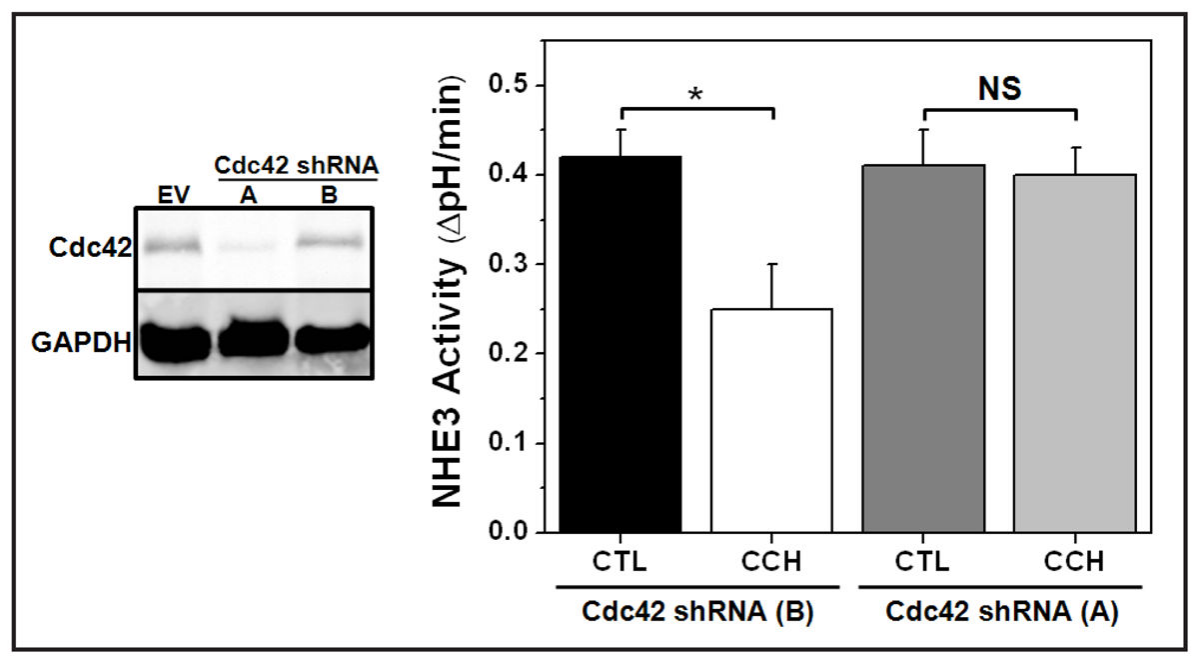
shRNA knockdown (KD) of Cdc42 in Caco-2/ BBe cells prevents carbachol inhibition of NHE3 activity. Western blot analysis of total cell lysates prepared from Caco-2/BBe cells stably transduced with lentivirus empty vector (EV) control or Cdc42 (left panel) shRNA constructs. Two different shRNA constructs (out of 5 total) are shown. shRNA A for Cdc42 represents the construct that demonstrated greatest degree of KD (84% compared to negative controls). shRNA B for Cdc42 did not exhibit any degree of KD compared to EV controls. Glyceraldehyde-3-phosphate dehydrogenase (*GAPDH*) was used as internal control. Stable Cdc42 KD Caco-2/BBe cells were treated with vehicle (*CTL*) or 10μM carbachol (*CCH*) for 5min. NHE3 activity was determined in the presence of 50 μM HOE-694 (concentration sufficient to inhibit all other NHE isoforms). CCH treatment resulted in a significant decrease of NHE3 activity in Cdc42 KD shRNA B cells but this effect was lost in Cdc42 KD shRNA A cells. Basal NHE3 activity in each line was not affected. *n* ≥ 3 for each condition. Rate of NHE3 activity (ΔpH/min) expressed as mean ± S.E. *p<0.05 compared to untreated controls. NS = Not significant.

Previous studies demonstrated that clathrin-independent endocytosis involves members of the Rho family of small GTPases [36]. Examples of clathrin-independent endocytosis that are dependent on small GTPases include CLIC/GEEC and macropinocytosis [21]. Since Cdc42 is required for some types of clathrin-independent endocytosis, we tested whether basal or CCH inhibition of NHE3 activity required Cdc42. In order to demonstrate that CCH signaling activates Cdc42, we measured the amount of GTP-bound Cdc42 (i.e. active form) after CCH treatment. After 5min CCH treatment, the levels of GTP-bound Cdc42 were significantly increased compared to untreated controls (Fig. 4). In fact, Cdc42 activation occurred as early as 1min post CCH and remained elevated for up to 20min (data not shown). We then pretreated Caco-2/BBe cells with the Cdc42 specific inhibitor, Pirl-1, and measured its effect on NHE3 activity. As shown in Figure 5, Pirl-1 treatment (20μM for 30min) prevented CCH inhibition of NHE3 activity, but did not affect basal activity. We further demonstrated a role for Cdc42 in CCH regulation using shRNA knockdown technology. We tested multiple shRNA probes and generated stable Cdc42 KD Caco-2/BBe cells (Fig. 6). By Western blot, Cdc42 KD (denoted as Cdc42 shRNA A) reduced expression by 74% compared to empty vector and ineffective Cdc42 shRNA constructs (Cdc42 shRNA B; Fig. 6). We then tested the effect of Cdc42 KD on NHE3 under basal and CCH treated conditions. As demonstrated in Figure 6, Cdc42 KD abolished CCH regulation of NHE3 activity but did not alter basal NHE3 activity when compared to Cdc42 shRNA KD B cells. These data support the hypothesis that CCH induced endocytosis of NHE3 requires lipid rafts and Cdc42.

### Inhibition of Cdc42, but not clathrin, prevents CCH-induced endocytosis of NHE3 in Caco-2/BBe cells

**Fig. 7:**
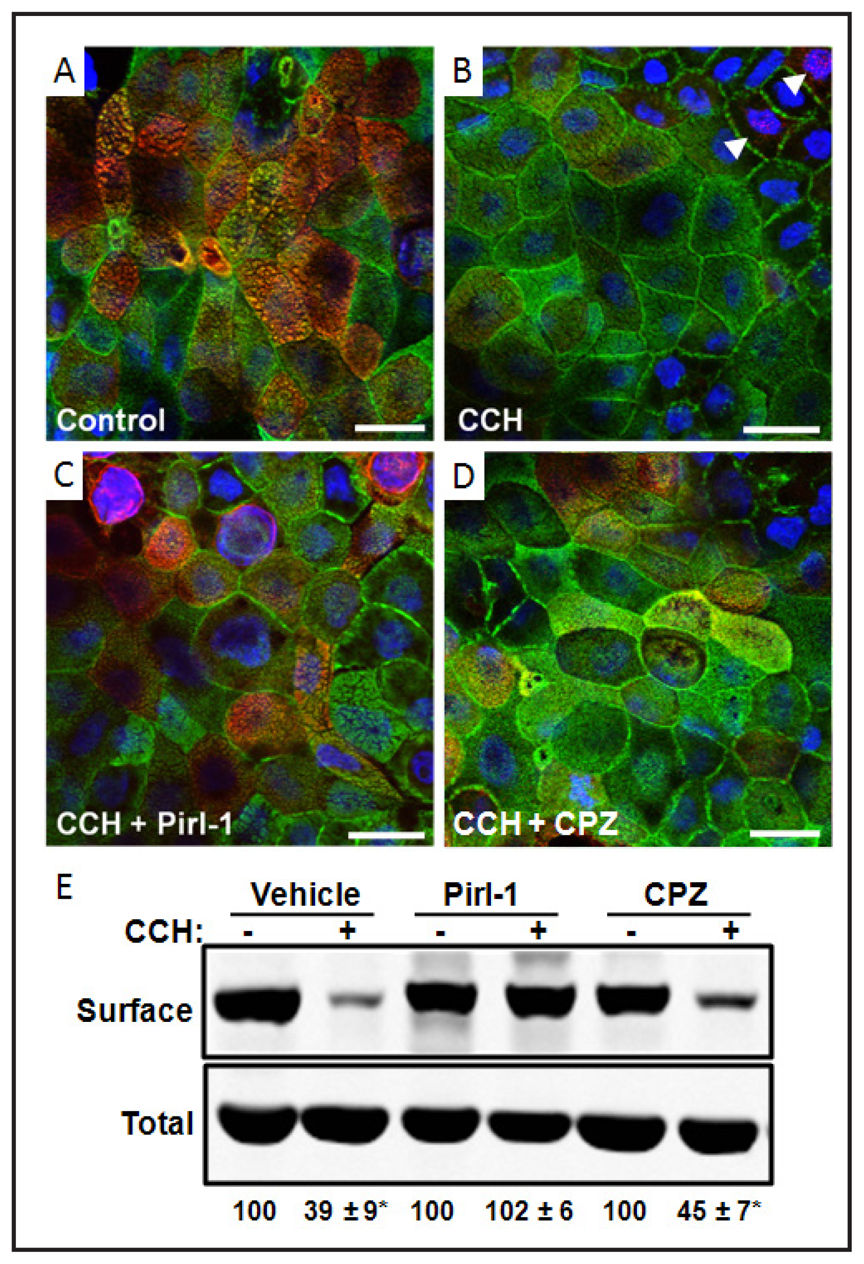
Inhibition of Cdc42, but not clathrin, prevent carbachol-induced endocytosis of NHE3 in Caco-2/BBe cells. Caco-2/BBe cells infected with 3HA-NHE3 adenovirus construct were treated with either vehicle (*control*) or 10μM carbachol (*CCH*) for 5min after pretreatment with either Pirl-1 or CPZ. After vehicle or CCH exposure, cells were fixed in 4% paraformaldehyde in PBS, permeabilized and stained for NHE3 (*red*; anti-HA antibody) and wheat germ aglutinnin (*green*; AlexaFluor 488 conjugated WGA) to mark BB. XY views are shown. *A*, NHE3 and WGA colocalize in the BB of untreated control cells. *B*, NHE3 co-localization with WGA and expression in the BB was reduced after CCH treatment. NHE3 appeared in intracellular vesicles below BB (arrowheads). *C*, Pretreatment of cells with Pirl-1 prevented CCH induced endocytosis of NHE3 while in *D*, CPZ treatment had no effect on the CCH induced changes in NHE3 localization and displayed similar localization to that in *B*. Scale bar = 20μm. Results were similar in 4 independent experiments. *E*, The effect of CPZ and Pirl-1 on CCH-mediated endocytosis of NHE3 was determined by surface biotinylation studies. Caco-2/BBe cells were treated with vehicle or CCH in the presence or absence of CPZ or Pirl-1 and surface biotinylated NHE3 was detected by Western blot. CCH decreased BB NHE3 levels, an effect that was lost when cells were pretreated with Pirl-1, but not CPZ. Surface NHE3 was normalized to total NHE3 in each treatment group and expressed as percent control. Numbers indicate percent change after CCH treatment compared to respective controls. Results were similar in five independent experiments. *p<0.05 compared to controls.

Since it is well established that CCH inhibition of NHE3 occurs by increased endocytosis, we performed immunofluorescence confocal microscopy to determine the localization of NHE3 under basal and CCH conditions. As shown in Figure 7A, NHE3 was localized to the BB of Caco-2/BBe cells similar to the localization of the BB marker, WGA, under basal conditions. After CCH treatment (Fig. 7B), the amount of NHE3 in the BB was reduced compared to WGA. Also, intracellular NHE3 vesicles (arrowheads) were observed in an area above the nucleus but below the BB after CCH treatment. We further demonstrated whether inhibition of clathrin or Cdc42 prevented CCH induced internalization of NHE3. In cells pretreated with Pirl-1, NHE3 colocalized with WGA in the BB indicating a block in endocytosis after CCH treatment (Fig. 7C). In contrast, NHE3 and WGA colocalization was lost in cells pretreated with chlorpromazine demonstrating that CCH-induced endocytosis of NHE3 occurred even though clathrin-mediated endocytosis was inhibited (Fig. 7D). In order to confirm that changes in BB NHE3 localization under these conditions, we performed surface biotinylation studies. In Caco-2/BBe cells treated with CCH, surface NHE3 expression was decreased by ~60% (Fig. 7E). This effect was lost in CCH treated cells pretreated with Pirl-1, suggesting that CCH-induced endocytosis of NHE3 involves Cdc42. Furthermore, CPZ treatment did not prevent CCH-mediated internalization of NHE3 further demonstrating that CME is not involved (Fig. 7E). These results also demonstrate that CCH activation of Cdc42 is necessary for clathrin-independent endocytosis of NHE3 in Caco-2/BBe cells. Since CCH signaling involves elevated [Ca^2+^]_i_, we next determined whether activation of Cdc42 by CCH was calcium dependent.

### CCH-induced activation of Cdc42 is Ca^2+^-dependent

**Fig. 8:**
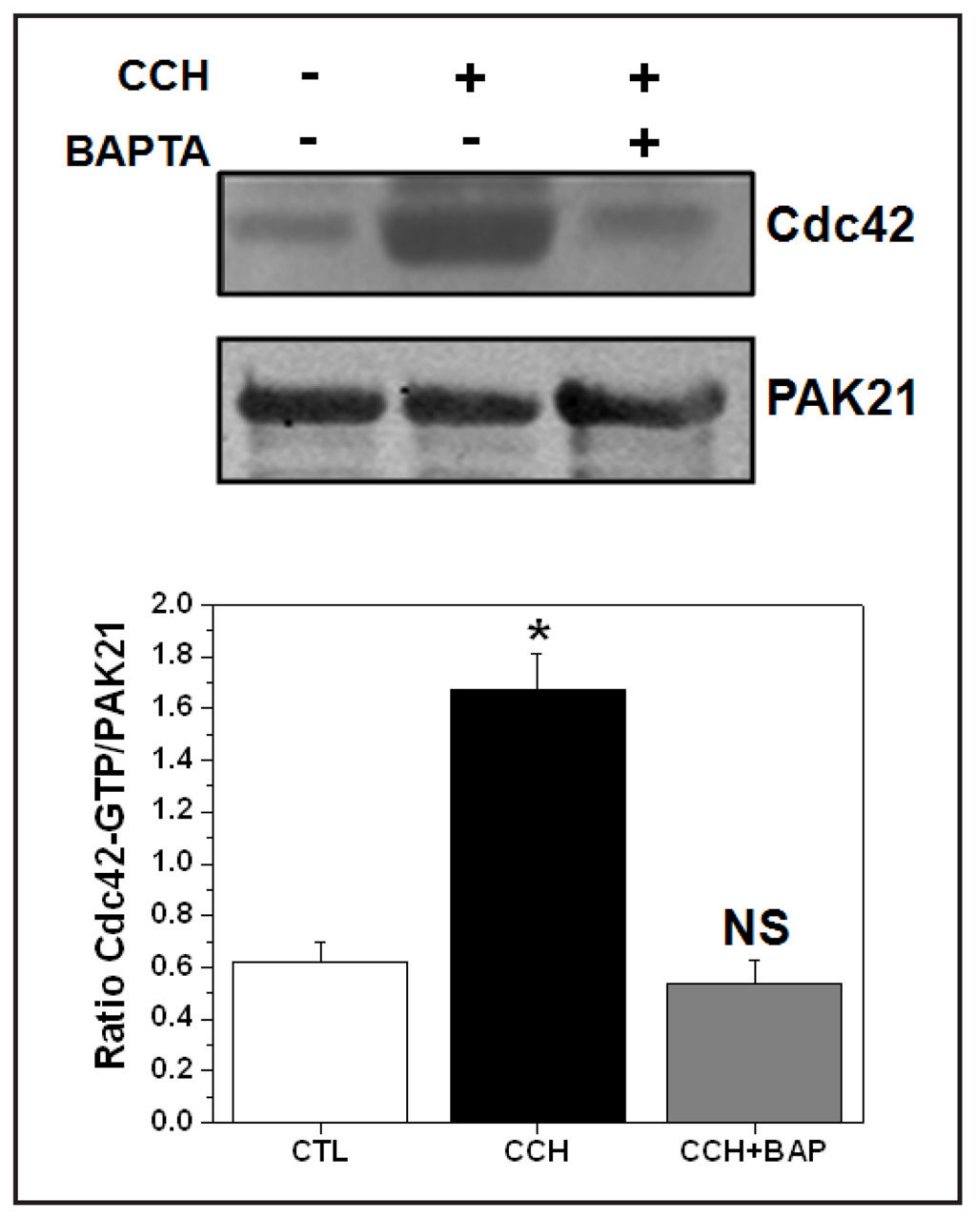
Carbachol activation of Cdc42 is calcium dependent. Caco-2/BBe cells were pretreated with vehicle (*CTL*) or 35μM BAPTA-AM (*BAP*) for 30 minutes prior to treatment with carbachol (*CCH*) for 5 min. Cells lysates from each condition were obtained and the amount of GTP-bound Cdc42 was quantified by Western blot analysis. CCH treatment increased the amount of GTP-bound Cdc42 compared to controls, an effect that was prevented by BAPTA-AM pretreatment. Blots are representative of results obtained from three independent experiments. Cdc42 protein levels normalized to PAK21 expressed as mean ± S.E. *p<0.05 compared to controls.

In order to demonstrate that CCH activation of Cdc42 depends on elevated [Ca^2+^]_i_, we measured the levels of GTP-bound Cdc42 in Caco-2/BBe cells pretreated with the [Ca^2+^]_i_ chelator, BAPTA-AM (35μM) prior to CCH treatment. As shown in Figure 8, CCH treatment (5min) increased the amount of GTP-bound Cdc42 compared to control conditions. Pretreatment with BAPTA-AM prevented CCH-induced increase in GTP-bound Cdc42 suggesting that CCH-induced increase in GTP-bound Cdc42 levels depend on elevation of [Ca^2+^]_i_. Taken together, the results of the current study demonstrate that CCH inhibition of NHE3 activity is due to increased endocytosis by a clathrin-independent mechanism that requires lipid rafts and Cdc42 in Caco-2/BBe cells.

## Discussion

This study provides additional insights into the trafficking mechanism involved in regulation of intestinal Na^+^ absorption under elevated [Ca^2+^]_i_ conditions. NHE3 is regulated by changes in its plasma membrane versus intracellular location as a result of alterations in the rates of endocytosis and/or exocytosis. NHE3 trafficking occurs under basal and acutely inhibited conditions (cAMP, cGMP, elevated [Ca^2+^]_i_) [1]. Moreover, previous studies have observed that constitutive and cAMP stimulated NHE3 endocytosis occur through a clathrin-dependent mechanism [17]. However, the mechanisms responsible for NHE3 trafficking in intestinal epithelial cells are not well understood. Recently, studies using confocal immunofluorescence microscopy and transmission electron microscopy (TEM) have characterized nine clathrin-independent endocytosis pathways in addition to clathrin-mediated endocytosis (CME) [21]. While previous studies have demonstrated that constitutive NHE3 endocytosis occurs via CME, our data demonstrates a role for clathrin-independent endocytosis in elevated [Ca^2+^]_i_ regulation of NHE3 activity. Our concept that NHE3 endocytosis may occur through multiple endocytic pathways is novel and our current study is the first to separate NHE3 endocytosis into clathrin-dependent and clathrin-independent pathways.

Recently, Musch et al demonstrated that synaptotagmin 1 regulates cAMP and Ca^2+^ mediated inhibition of NHE3 activity in a clathrin-dependent manner [17]. In their study, the effect of elevated [Ca^2+^]_i_ on NHE3 trafficking was determined using thapsigargin, which was used to elevate cytoplasmic Ca^2+^ by preventing reuptake of released Ca^2+^ into intracellular stores. This study concluded that Ca^2+^ stimulated endocytosis of NHE3 occurred in a similar manner to that of cAMP. While this study evaluated the downstream effect of elevated [Ca^2+^]_i_ in stimulating NHE3 endocytosis, the study did not determine whether ligand-receptor mediated Ca^2+^ signaling regulates NHE3 endocytosis in a similar manner. We have previously demonstrated in the intact small intestine that carbachol (CCH) signaling, which elevates apical [Ca^2+^]_i_ via basolateral membrane (BLM) muscarinic receptors (M3), inhibited NHE3 activity by stimulating endocytosis [2–5, 14]. However, elevation of [Ca^2+^]_i_ using the calcium ionophore, A23187, alone was not able to duplicate all of the effects of BLM M3 cholinergic signaling [37]. These results emphasize the importance of using physiologically relevant agents to study the mechanisms responsible for Ca^2+^ inhibition of NHE3 activity in intestinal epithelial cells.

The results from the current study are the first to demonstrate that CCH inhibition of NHE3 activity is entirely lipid raft dependent. NHE3 exists in multiple pools in the BB of intestinal and renal epithelial cells; however, the regulation of NHE3 in these different pools has only been partially described [15, 16]. In the current study, we demonstrate that basal NHE3 activity is increased after MβCD treatment in Caco-2/BBe cells (Fig. 3). These results suggest that a pool of NHE3 resides in lipid rafts under basal conditions and participates in constitutive recycling. A role for lipid rafts has been suggested in both intestinal and renal epithelial cells; although, NHE3 is not believed to undergo stimulated endocytosis in renal proximal tubule cells [6, 38–41]. In the intestine, CCH stimulates rapid translocation of signaling proteins to detergent resistant membrane fractions that include lipid rafts [13]. In fact, pretreatment of the rabbit ileum with MβCD prior to CCH, prevented the increase in the size of NHE3 containing multi-protein complexes that result from CCH signaling [13]. Based on these findings, it is possible that disruption of lipid rafts may prevent organization of signaling complexes that are required for CCH-mediated endocytosis. However, the mechanism by which lipid rafts contribute to inhibition of NHE3 activity remains to be determined.

It has been recently demonstrated that clathrin-independent endocytosis requires lipid rafts while clathrin-mediated endocytosis does not. Since several types of clathrin-independent endocytosis pathways have been described, the goal of the current study was to separate the contributions of clathrin-dependent and clathrin-independent endocytosis to basal and CCH inhibition of NHE3 activity. Our results show that basal, but not CCH-inhibited, NHE3 activity involves clathrin-mediated endocytosis based on studies using chlorpromazine and K^+^-depletion to prevent CME. Although all inhibitors of CME have been shown to elicit non-specific effects on trafficking, our results support previous studies that constitutive NHE3 endocytosis is clathrin-dependent [17, 23]. Whether additional CME inhibitors affect basal NHE3 trafficking in a similar manner to CPZ and K^+^-depletion remains to be determined. Our results also demonstrate a modest (~30%) increase in basal NHE3 activity after MβCD treatment suggesting that a portion of BB NHE3 resides in lipid rafts under basal conditions and is inhibitory of basal NHE3 activity in Caco-2/BBe cells. These data also support previous studies in the intact rabbit ileum and in renal proximal tubule cell lines that demonstrate a role for lipid rafts in regulating basal NHE3 activity [15, 16]. However, under elevated [Ca^2+^] _i_ conditions, NHE3 regulation is completely lipid raft dependent. These results contrast with previous studies in which calcium ionophores or thapsigargin were used to elevate [Ca^2+^]_i_. In these studies, CME was responsible for NHE3 endocytosis. In this study, we administered CCH to mimic the neurohumoral regulation of normal digestion. CCH binds to basolateral muscarinic (M3) receptors to elicit their physiological effects. Recent studies have linked CCH signaling to stimulation of clathrin-independent endocytosis; however, more detailed studies of the signaling molecules involved in this pathway are necessary [42].

Several types of clathrin-independent endocytosis have been described to require small GTPases including Cdc42, which has been shown to play an integral role in CLIC/ GEEC and macropinocytosis [21]. Our results are the first to demonstrate a role for Cdc42 in NHE3 regulation. Here we show that CCH inhibition of NHE3 activity is also entirely Cdc42 dependent. Furthermore, we demonstrate that activated Cdc42 is required for CCH inhibition of NHE3 activity and that activation of Cdc42 occurs rapidly (~1min) after CCH treatment. This rapid signaling is consistent with previous studies demonstrating rapid activation of other CCH signaling events including activated PLC-γ and PKC at the apical membrane in intestinal epithelial cells. Cdc42 is well established to play an integral role in actin remodeling events that are necessary for endocytic vesicle formation and vesicle trafficking. These results in combination with our lipid raft results suggest that CCH-induced endocytosis of NHE3 involves a clathrin-independent pathway. However, identification of the type of clathin-independent pathway involved is not known. Furthermore, more detailed studies about the role of Cdc42 in actin remodeling under CCH treated conditions are necessary to characterize the type of clathrin-independent endocytosis that occurs under these conditions.

Why does NHE3 traffic through clathrin-dependent and clathrin-independent pathways? Recent studies have suggested that membrane cargo can internalize through multiple endocytic pathways [43]. These studies also demonstrated that different endocytic pathways (i.e. clathrin-dependent versus clathrin-independent) may lead to separate intracellular destinations including recycling endosomes versus lysosomes/proteosomes [44]. Other studies have demonstrated that different membrane cargo internalized by either clathrin-dependent or clathrin-independent pathways can merge and share similar intracellular endocytic compartments, including early and recycling endosomes [43]. It has been shown that CCH treatment results in the increased association of NHE3 with EEA1-positive endosomes [13]. A recent report has observed that clathrin-independent endocytosis directs MHC-I trafficking through EEA1 endosomes and eventually leads to delivery to lysosomes for degradation [38]. Whether NHE3 protein expression is downregulated in response to CCH treatment or whether other NHE3 trafficking involves alternate endosomal compartments remains to be determined.

The results of this study demonstrate that CCH inhibition of NHE3 activity requires lipid rafts and activated Cdc42 and suggests that NHE3 inhibition involves a clathrin-independent endocytosis pathway. Whether this pathway is similar to a clathrin-independent pathway that has been previously described or represents a novel variation remains to be determined. Furthermore, we demonstrate that NHE3 regulation by endocytosis occurs through multiple pathways and suggests a higher order of second messenger regulation than previously recognized. The results of our study may provide insight into how Na^+^ absorption is regulated under the elevated calcium conditions observed under normal digestion and in some acute diarrheal diseases.
